# Clinical study on the treatment of moderate to severe persistent allergic rhinitis by posterior nasal nerve combined with anterior ethmoid neurotomy

**DOI:** 10.12669/pjms.38.7.5561

**Published:** 2022

**Authors:** Rongrong Wu, Lvhua Dong, Huajie Mao, Jianjun Wang, Dijiang Ma, Jianing Sun

**Affiliations:** 1 Rongrong Wu, Department of ENT and Head & Neck Surgery, Linhai Second People’s Hospital, Taizhou 317016, Zhejiang, China; 2 Lvhua Dong, Department of ENT and Head & Neck Surgery, Linhai Second People’s Hospital, Taizhou 317016, Zhejiang, China; 3 Huajie Mao, Department of ENT and Head & Neck Surgery, Linhai Second People’s Hospital, Taizhou 317016, Zhejiang, China; 4 Jianjun Wang, Department of ENT and Head & Neck Surgery, Linhai Second People’s Hospital, Taizhou 317016, Zhejiang, China; 5 Dijiang Ma, Department of ENT and Head & Neck Surgery, Yuyao People’s Hospital of Zhejiang Province, Yuyao 315400, Zhejiang, China; 6 Jianing Sun, Department of ENT and Head & Neck Surgery, Yuyao People’s Hospital of Zhejiang Province, Yuyao 315400, Zhejiang, China

**Keywords:** Posterior nasal nerve, Anterior ethmoid neurotomy, Moderate to severe persistent allergic rhinitis

## Abstract

**Objectives:**

To investigate the clinical effect of posterior nasal nerve combined with anterior ethmoid neurotomy in the treatment of moderate to severe persistent allergic rhinitis.

**Methods:**

Thirty patients with moderate to severe persistent allergic rhinitis admitted to Linhai Second People’s Hospital from August 2019 to June 2020 were selected as subjects for prospective study and design. All patients underwent posterior nasal neurotomy and anterior ethmoid neurotomy simultaneously. Subsequently, the efficacy of all patients at 0.5 and one year postoperatively was compared. Their symptom score preoperatively and one year postoperatively as well as their preoperative and postoperative quality of life score were compared, and related adverse reactions were collected.

**Results:**

The curative effect ratio was 60% after 0.5 years and 90.0% after one year, showing a significant increase (*χ^2^*=12.000, P=0.007<0.05). The symptom score at one year postoperatively (1.15±0.32) was lower than that preoperatively (2.12±0.58), with a statistically significant difference (t=11.351, P=0.000<0.05); In terms of quality of life, nasal symptoms, ocular symptoms, practical difficulties, sleep disorders, emotional disorders and other symptoms were lower than those preoperatively, with statistically significant differences (P<0.05). Adverse reactions occurred in 4 patients (13.33%), but were eliminated after treatment.

**Conclusion:**

Posterior nasal nerve combined with anterior ethmoid neurotomy is a safe and reliable surgical method for the treatment of moderate to severe persistent allergic rhinitis, boasting a variety of benefits such as symptomatic relief, improved quality of life, and reduced adverse reactions, which is worthy of clinical promotion.

## INTRODUCTION

Allergic rhinitis, as a common otorhinolaryngologic disease, occurs at any age and has a complicated pathogenesis.^1^ It occurs all over the world with an increasing incidence in recent years. It has been pointed out in relevant studies that allergic rhinitis is a chronic non-infectious inflammation, and in severe cases, it will affect the daily life and work of patients.^2^ Vidian neurectomy is a common surgical method in the treatment of allergic rhinitis, with an effective rate of about 76.0%. However, its clinical application is subject to certain limitations because of the large trauma left, resulting in patients suffering from varying degrees of ocular dry complications.^3^-^5^ For this reason, the priority of clinical study is gradually put on the improvement of vidian neurotomy, and the combined treatment method is favored by first-line clinicians.

The combined treatment method is to combine the inferior turbinate, posterior superior nasal nerve, anterior ethmoidal nerve and other neurotomy, which breaks through the limitations of single vidian neurotomy. Posterior nasal neurotomy is to cut off the postganglionic fibers of the sphenopalatine ganglia and the sensory fibers of the maxillary nerve, but does not include the parasympathetic nerve of the lacrimal gland. The anterior ethmoidal nerve has the sensory nerve fibers of the first trigeminal nerve and oculomotor nerve nucleus. The combination of the two surgeries can effectively relieve the symptoms of patients and achieve preferable clinical therapeutic effect.^6^-^8^ The specific details are reported as follows.

## METHODS

Thirty patients with moderate to severe persistent allergic rhinitis admitted to Linhai Second People’s Hospital from August 2019 to June 2020 were selected as subjects for prospective study and design. The sample size was calculated by the formula 
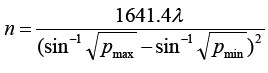
.There were 17 males (56.7%) and 13 females (43.3%), aged from 13 to 81 years, with an average age of (51.18±19.22) years. Their BMI ranged from 17.49 to 29.81 kg/m^2^, with an average BMI of (22.94±3.41) kg/m^2^. The course of disease was 5-12 years, with an average course of disease of (8.45±2.37) years. All patients underwent posterior nasal neurotomy and anterior ethmoid neurotomy simultaneously. And all patients were performed by surgeons from the same medical team.

### Ethical Approval

The study was approved by the Institutional Ethics Committee of Linhai Second People’s Hospital on February 5, 2020 (No.[2020]12), and written informed consent was obtained from all participants.

### Inclusion criteria:

- Patients who meet the diagnostic criteria of “Guidelines for the Diagnosis and Treatment of Allergic Rhinitis” (2015 edition)^9^;
- Patients with moderate to severe persistent allergic rhinitis by skin prick test;
- Patients with varying degrees of runny nose, nasal congestion, nasal itching and other symptoms;
- Patients who were informed of this study and signed an informed consent form

### Exclusion criteria:

- Patients with nasal polyps, sinusitis, nasal cavity tumors and other diseases;
- Patients complicated with severe intestinal, liver, kidney, heart, lung and coagulation diseases;
- Patients with other acute and chronic diseases;
- Patients with severe mental illness.

### Posterior nasal neurotomy under nasal endoscope

All patients underwent general anesthesia and routine intubation. After successful anesthesia, cotton pads soaked in adrenaline saline were used to shrink the nasal mucosa. Under nasal endoscope, a longitudinal incision was made at 0.5cm in front of the tail of the middle turbinate attachment, and the nasal mucosa was cut to the bone surface. The mucosa was peeled posteriorly along the vertical plate of the palatine bone to expose the ethmoidal crest. A rongeur was used to bite the sieve crest to expose the nerve blood separated from the sphenopalatine foramen and identify the course and branch of the posterior nasal nerve. A monopolar electric knife was used to incise the vascular nerve bundle until the bony surface around the sphenopalatine foramen was exposed, and the sphenoid process of the palatine bone was removed with a nucleus pulposus forceps to expose the palatal sheath. Subsequently, the palatine artery and the medial palatine sheath were cut, the broken end was separated outwardly and upward, and the pterygous canal nerve was exposed until it was completely cut off. After the surgical cavity was fully hemostasis, the mucosa was reduced, and the sponge was removed 2-3 days after the surgical package was inflated. Postoperatively, maintenance treatment with related drugs was applied to promote recovery.

The anterior ethmoidal nerves of the lateral nasal branch and septal branch of the nasal cavity were cut off with a plasma treatment needle. Below 30°, the mucous membrane of the posterior lateral wall of the nasal colliculus was cut down to the bone surface using the needle of the nasal endoscope plasma therapy instrument. The contralateral side was then treated in the same way, with an arc cut line and a length of 2cm.

Multi-dimensional scores were used to compare the preoperative and postoperative symptom scores of all patients, and the efficacy of 0.5 years and one year was confirmed. The clinical symptoms are mainly based on the symptom score. one point means that the patient sneezes three to five times at a time, blows nose four times or less, and feels nasal congestion and intermittent itching. 2 points means the patient sneezes 6-10 times at a time, blows nose 5-10 times, with intermittent nasal congestion and obvious itching, but can be tolerated; three points means the patient sneezes more than 10 times at a time, blows nose more than 10 times, has obvious nasal congestion, obvious itching, significant nasal ant sensation, and needs to breathe through his mouth. Furthermore, the preoperative and postoperative quality of life scores were compared based on the “Rhinoconjunctivitis Quality of Life Questionnaire (RQLQ)”, which mainly included nasal symptoms, eye symptoms, practical difficulties, sleep disorders, and emotional disorders, with a total of 28 items. Each item corresponds to 0-6 points, among which three items can be selected for the biggest confusion in life, and the rest are 4 items for nasal symptoms, four items for eye symptoms, three items for practical difficulties, 3 items for sleep disorders, four items for emotional disorders, and seven items for other symptoms.

### Statistical Methods

SPSS25.0 statistical software was used for statistical analysis. Measurement data were expressed as mean ± standard deviation (*x̅*±*s*), paired sample T test was used for preoperative and postoperative comparison of patients, counting data were expressed as n (%), and Chi-square X2 test was used. P<0.05 indicates a statistically significant difference.

## RESULTS

The curative effect ratio was 60% after 0.5 years and 90.0% after 1 year, showing a significant increase (*χ^2^*=12.000, P=0.007 <0.05). Table-I. Symptom scores, nasal symptoms, eye symptoms, practical difficulties, sleep disorders, emotional disorders, and other symptoms at one year postoperatively were lower than those preoperatively, with statistically significant differences (P<0.05. Table-II.

**Table I:** Efficacy analysis.

Item	Efficacy after 6 months		Efficacy after 1 year		χ^2^	P

	n	Percentage	n	Percentage		
Invalid	12	40.00%	3	10.00%	12.000	0.007
Effective	10	33.33%	13	43.33%		
Markedly effective	8	26.67%	14	46.67%		
Total	30	100.00%	30	100.00%		
Effective rate	18	60.00%	27	90.00%		

**Table II:** Symptom score and quality of life score and their paired tests.

Name		Pairing (Mean ± standard deviation)		Difference (pair 1- pair 2)	*t*□	*p*□

		Preoperatively	1 year preoperatively			
Symptom score		2.12±0.58	1.15±0.32	0.97	11.351	0.000**
Quality of life	Nasal symptoms	19.47±4.39	10.34±2.14	9.13	14.166	0.000**
	Ocular symptoms	18.47±4.16	7.68±1.38	10.79	17.037	0.000**
	Practical difficulties	14.34±2.34	5.39±2.14	8.95	25.812	0.000**
	Sleep disorders	14.68±2.16	6.47±1.58	8.21	21.454	0.000**
	Emotional disorders	18.67±4.35	8.67±2.16	10.00	15.748	0.000**
	Other symptoms	28.69±6.24	16.69±4.68	12.00	10.811	0.000**

*P<0.05

** *P*<0.01

There were four cases (13.33%) of postoperative adverse reactions, including one case of vomiting, one case of malignancy and two cases of hypotension. However, after follow-up treatment and nursing, all four cases of adverse reactions were eliminated.

## DISCUSSION

Allergic rhinitis, as a clinically thorny problem, is a complex inflammatory invasion caused by the invasion of specific strygens, eosinophils, IgE intervention and other factors, which damage the trigeminal and ethmoidal nerves. Relevant studies have pointed out that allergic rhinitis is mostly caused by antigen-antibody reactions, and the three symptoms of runny nose, sneezing, and nasal congestion have a close bearing on sensory nerves connected with nasal mucosa and related vegetative nerves.^10^ External factors stimulate the trigeminal nerve endings to induce nerve impulse, making the excitement spread to the center, and then induce sneezing through the high spinal cord. Excessive excitement of the parasympathetic nerve can also bring about uncontrolled glandular secretion, abnormal excitement and vasodilation, eventually leading to nasal congestion and runny nose. For this reason, some scholars believe that cutting off the vidian nerve that controls the nasal mucosa can effectively control the runny nose and nasal congestion induced by allergic rhinitis. The posterior superior nasal nerve belongs to the posterior nerve fiber of the vidian nerve and the sensory fiber of the maxillary nerve, and cutting this part of the nerve has a similar effect with the vidian nerve, both of which can relieve the symptoms of patient 11 Cutting the posterior superior nasal nerve can also avoid eye dryness, while cutting the vidian nerve may easily cause dry eyes by destroying the parasympathetic nerve that innervates the lacrimal gland. It has been pointed out in relevant studies that the anterior ethmoid nerve is the nerve fiber of both the trigeminal nerve and the oculomotor nerve. If this part of the nerve is cut, the symptoms of runny nose, sneezing and stuffy nose can be relieved, and the patient’s curative effect can be significantly improved. The principal predominance of the combination of the two surgeries are mainly reflected in the following points: First, trigeminal nerve endings can be effectively cut off to block the transmission of neurons caused by stimulation to induce sneezing; Second, the occurrence of runny nose can be reduced by cutting off the parasympathetic nerve fibers; Third, the volume of inferior turbinate can be reduced to improve nasal cavity shape effectively.

In the treatment of atopic rhinitis, priority should be given to both inflammation and nasal structural abnormalities, while seasonal and allergens should be considered to develop targeted programs; For patients with persistent allergic rhinitis, abnormal nasal structure will lead to changes in nasal airflow dynamics, leading to hypersensitivity of nasal mucosa.^12^,^13^ Among them, the three symptoms of sneezing, runny nose, and nasal congestion are all related to the nasal mucosa-related nerve imbalance, that is, the imbalance between the trigeminal nerve endings and the parasympathetic nerve, which eventually induce typical symptoms. In this experiment, significant differences can be seen in the preoperative and postoperative symptom scores, and the postoperative symptom scores were significantly reduced because the relevant nerve was cut off after the posterior nasal nerve combined with anterior ethmoid neurotomy to avoid nerve impulse-induced symptoms. According to the “Rhinoconjunctivitis Quality of Life Questionnaire (RQLQ)”, both nasal and ocular symptoms were ameliorated, indicating that combined surgery can effectively improve the patient’s symptoms and reduce the occurrence of dry eye symptoms; Meanwhile, sleep disorders, emotional disorders, practical difficulties, and other symptoms were improved, further improving the quality of life of patients, indicating that combined surgery is touted to improve the quality of life of patients while improving their symptoms. Studies have pointed out that nasal neurotomy has a significant effect on the treatment of severe allergic rhinitis, boasting that it can reduce patients’ pain and improve their quality of life without complications^14^,^15^, which is consistent with this study to a certain extent. Definitely, when performing nasal neurotomy, consideration should also be placed on whether the patient has related comorbidities, and the impact of the degree of allergies on the surgical efficacy should not be ignored. It has been unanimously agreed in many overseas studies that allergic rhinitis is a scientific problem, and epidemiological investigation should be conducted from the aspects of pathogens, allergens and inflammatory reactions to explore the influencing factors, diagnosis, treatment and prevention methods, so as to effectively reduce the occurrence of allergic rhinitis.^16^-^18^ It has also been pointed out that the treatment of allergic rhinitis is not limited to nerve blocking, but can also be treated by saline irrigation, allergen immunotherapy and other methods to promote the early recovery of patients.^19^-^21^

### Limitations of this study

The number of subjects included in this study was limited, so the conclusions drawn may not be very convincing. In addition, this study used intra-group before and after control, and no other control group was set. We will further design relevant randomized controlled trials in the future study to verify this conclusion.

## CONCLUSION

Posterior nasal nerve combined with anterior ethmoid neurotomy is a safe and reliable surgical method for the treatment of moderate to severe persistent allergic rhinitis, boasting a variety of benefits such as symptomatic relief, improved quality of life, and reduced adverse reactions.

### Authors’ Contributions:

**RW** & **JS:** Designed this study, prepared this manuscript, are responsible and accountable for the accuracy and integrity of the work;

**LD & HM:** Collected and analyzed clinical data;

**JW** & **DM:** Data analysis, Significantly revised this manuscript.
